# Simultaneous Periprosthetic Acetabular Fracture and Contralateral B-Type Compression Injury of the Pelvic Ring: A Case Report of a Rare Injury Combination

**DOI:** 10.1155/2013/607046

**Published:** 2013-10-09

**Authors:** Sven Märdian, Klaus-Dieter Schaser, Florian Wichlas, Philipp Schwabe

**Affiliations:** Center for Musculoskeletal Surgery, Charité University Medicine Berlin, Augustenburger Platz 1, 13353 Berlin, Germany

## Abstract

The periprosthetic fracture of the acetabulum is a rare injury, and its management is only sporadically reported in the literature. A few case reports are available which mainly focus on periprosthetic acetabular fractures in the elderly population. In our case, a 32-year-old patient suffered from a periprosthetic acetabular fracture in combination with a pelvic ring injury following a high velocity accident. The fracture morphology allowed a salvage of the prosthesis with an open reduction and internal fixation with a good radiographic and functional outcome one year after trauma.

## 1. Introduction

Almost 200.000 total hip replacements (THAs) are performed in Germany [1] annually, and even higher numbers are reported for the United States, which account for $9.2 billion in hospitalization costs per year [2]. The main indication for arthroplasty is osteoarthritis in predominantly the elderly population. Due to the demographic changes in the western industrial countries with an aging but active population, the continuously prolonged life expectancy, and the increasing survival time of the prostheses, the incidence and rate of periprosthetic fractures are further expected to markedly grow [3]. According to the data of Lindahl et al. [4] periprosthetic fractures count for 9.5% of all revision surgeries and are the third frequent cause for revision in arthroplasties. As there are much data concerning periprosthetic femoral fractures, only little information is available about pelvic and periacetabular fractures in the presence of preexisting THA [5]. The literature available on that issue considers only elderly patients, whereas no publication deals with young patients who suffered from that type of injury. We report our management of a patient with a unique injury combination: a posterior column periprosthetic acetabular fracture with a pelvic ring fracture sustained after a fall from great height. 

## 2. Case Report

**Figure 1:**
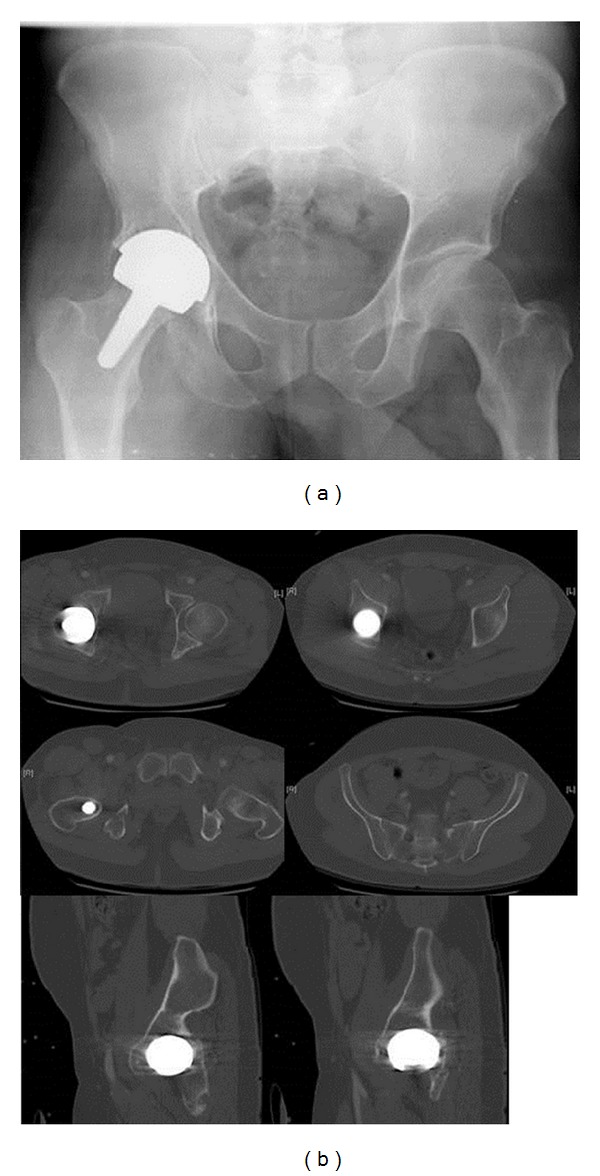
The patient presented with a pelvic ring compression fracture at the right side as well as a periprosthetic posterior column fracture at the left side (emergency X-ray and multislice CT scan).

**Figure 2:**
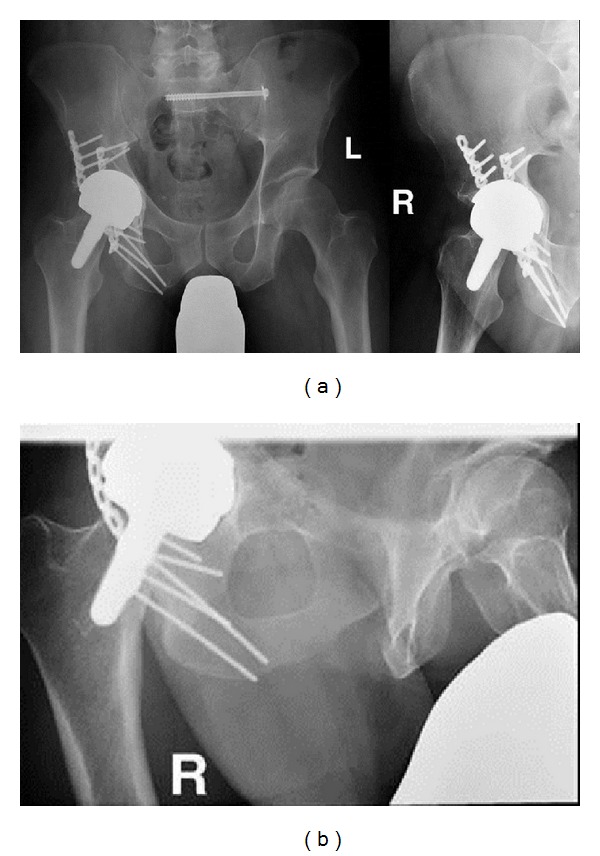
Postoperative X-ray 1 year after trauma showing bony healing in an anatomic position.

A 32-year-old male patient sustained multiple injuries after a fall from six meters while being trapped in a crashing elevator. After the rescue by the emergency medical team, he was immediately transferred to our level I trauma center and treated according to ATLS guidelines. At presentation in the emergency room, the patient was hemodynamically stable with a GCS of 15. He complained about back pain as well as pain in both hips. The physical examination revealed moderate instability of the pelvic ring as well as pain and crepitation during passive motion of the right hip. A pelvic binder was applied in the emergency room to primarily stabilize the pelvic ring. Further workup with X-rays and a multislice CT scan demonstrated a thoracic spine (T_12_) fracture (no neurological deficits, type 51-A1.1 according to AO), a B-type compression fracture of the left pelvic ring (61-B2.1 b_2_, c_1_ according to OTA), and a periprosthetic posterior column fracture of the right acetabulum (62-A2.3 a_1_ according to OTA, Figures 1(a)-1(b)). In his medical history, the patient suffered from an adenoma of the pituitary gland which was resected in 2008. Due to long-term corticoid medication during that course of treatment the patient developed a femoral head necrosis in 2010 which was treated by another institution with a total hip replacement in 2011 (midhead resection and resurfacing arthroplasty; BMHR prosthesis according to McMinn). Since the preoperative radiological diagnostics showed no signs of loosening of the acetabular cup, the posterior column and the posterior wall were stabilized via the Kocher-Langenbeck approach after open reduction with two 3.5 mm titanium reconstruction plates (Figure 2). Accordingly, the intraoperative assessment of the acetabular cup showed a firm grip in the anterior column. Therefore, we classified the fracture as type I after Peterson II and Lewallen [6]. Fracture reduction and fixation could be performed anatomically, and the acetabular reconstruction with the two plates resulted in fixation of adequate stability. No bone grafting was required as the bone quality appeared to be excellent due to the patient's young age. After completing this first part of the operation which was done in a lateral position, the patient was repositioned in a supine position and the contralateral posterior pelvic ring was addressed with a percutaneous sacroiliac lag screw. This procedure was performed using a 3D fluoroscopy based navigation system (ARCADIS Orbic 3D by Siemens, BrainLab navigation system).

The patient was mobilised with limited weight bearing (15 kg partial weight bearing) and limitation of flexion (60°) for the right hip and full weight bearing on the left side for 12 weeks. Concerning the spine injury, a conservative approach was pursued with mobilisation in a three-point corsage for 12 weeks. During the postoperative course, the patient was regularly seen in the outpatient department and showed an uneventful healing. 

**Figure 3:**
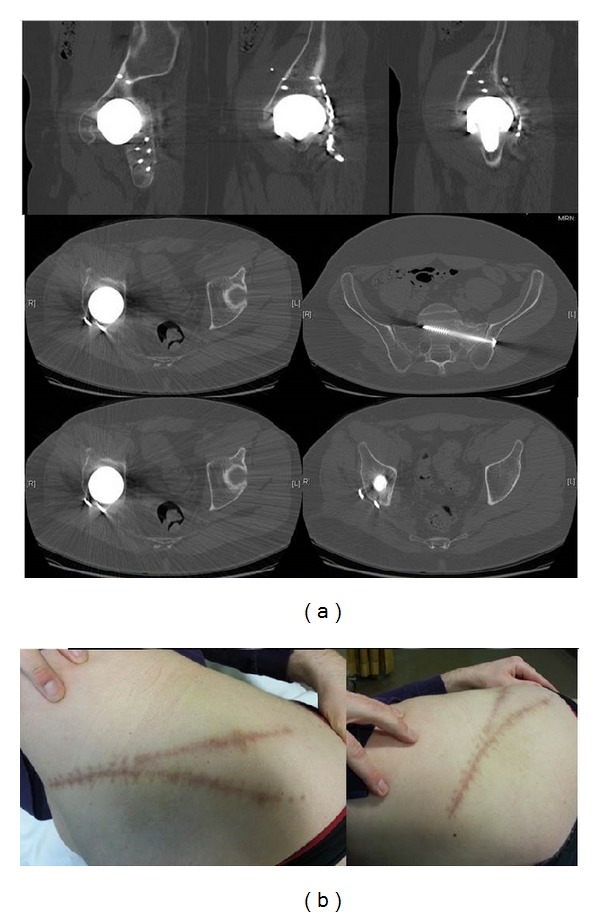
Clinical wound condition and CT-scan result 1 year postoperatively.

At the 12-month follow-up (see Figures 2 and 3) visit, the patient presented with inconspicuous scars and pain-free mobilisation without limping. The range of motion of the right hip showed 100° flexion and 10° of extension (0°/0°/130° left side). External and internal rotation showed a free range of motion with 45°/0°/35° for both hips. The patient's hip range of motion and general activity were identical to the level prior to the accident, and he achieved 94 points in the Harris Hip Score. The neurovascular status was intact on both sides. The radiographs as well as the CT scan showed a complete bony union with a well integrated acetabular cup. No signs of component loosening or heterotopic ossifications could be detected. The patient was doing well and had been back to work for 6 months already. 

## 3. Discussion

The available literature concerning periprosthetic fractures after total hip replacement mainly concentrates on periprosthetic fractures of the femur which is much more often affected than the acetabulum [2]. Sufficient data and management strategies, all based on the location/type of the fracture, the status of the host bone, and the stability of the femoral component, have extensively been published [7–9]. However, periprosthetic acetabular fractures are rare and, available literature is limited. The largest case series has been published by Peterson II and Lewallen in 1996 [6]. They presented only eleven cases of patients being over 60 years of age with periprosthetic acetabular fractures. Four were of traumatic nature, and only two were classified as unstable. None of the cases occurred due to a high energy accident. Their results show a poor prognosis with regard to the survival of the acetabular component even in stable situations. Although based on few cases only, it is the only classification available in the literature.

The presented case is unique since it shows a young patient with a periprosthetic acetabular fracture in combination with an unstable posterior pelvic ring injury. This combination of injuries in young patients is fundamentally rare due to the fact that mainly elderly patients already underwent a THA. However, this patient group is, due to their higher level of activity, at higher risk to sustain a high energy trauma causing those injuries. Therefore, the treatment strategy gets more complicated especially in terms of decision making (reconstruction versus revision arthroplasty). The main question that remains is the stability of the components. In our case sings of loosening were absent, and in our opinion a conservative approach would have implied a higher risk for secondary loosening of the acetabular cup because of the impaired alignment and bony stability of the dorsal acetabular rim. So we chose a reconstructive course. The pelvic ring instability was stabilized in order to achieve a stable situation in which the patient can perform full weight bearing at least at one side. Performing a revision arthroplasty would have implied a revision of the femoral component as well as a significant bony defect in the acetabulum in case of a loose acetabular component. Referring to the ongoing discussion about MoM THAs [10], we did not see an indication to replace the implanted MoM THA because the patient was free of pain and had no change in his general health status prior to the accident. Regarding the upcoming revision arthroplasties a young patient has to deal with in the future, a reconstruction to obtain the bone stock is preferable in our opinion in cases of absent signs of loosening. In case of a loose acetabular component intraoperatively, the revision arthroplasty could have been performed through the utilised posterior approach so that the final decision concerning reconstruction or revision arthroplasty could be made intraoperatively. However, in such cases all possibilities of reconstructional options as well as appropriate revision arthroplasty implants must be available on site. Our strategy led to a good functional result and the ability to get back to work without restrictions within 6 month after trauma. 

The main question of the treatment decision that still remains is the condition of the prosthetic component. In cases of loosening an osteosynthetic approach will fail. Therefore, we recommend a reconstruction instead of revision arthroplasty in cases of periprosthetic acetabular fractures with stable prosthetic components in young patients.

Preoperative planning should, among other facts, focus on the choice of approach. If loosening of the acetabular component cannot be prevented, the revision arthroplasty should be possible through the same approach. Expertise and suitable implants should be in readiness for such situations.
